# Morphology and Mechanics of Star Copolymer Ultrathin Films Probed by Atomic Force Microscopy in the Air and in Liquid

**DOI:** 10.3390/ma17030592

**Published:** 2024-01-25

**Authors:** Cristiano Albonetti, Lorella Izzo, Giovanni Vigliotta, Matilde Sublimi Saponetti, Fabiola Liscio, Fabrizio Bobba

**Affiliations:** 1 Consiglio Nazionale delle Ricerche, Istituto Per lo Studio dei Materiali Nanostrutturati (CNR-ISMN), Via P. Gobetti 101, 40129 Bologna, Italy; 2 Consiglio Nazionale delle Ricerche, Istituto Superconduttori, Materiali Innovativi e Dispositivi (CNR-SPIN), Via Giovanni Paolo II, 132, 84084 Fisciano, SA, Italy; 3 Dipartimento di Biotecnologie e Scienze della Vita, Università degli Studi Dell’insubria, Via J.H. Dunant, 3, 21100 Varese, Italy; lorella.izzo@uninsubria.it; 4 Dipartimento di Chimica e Biologia “A. Zambelli”, Università degli Studi di Salerno, Via Giovanni Paolo II, 132, 84084 Fisciano, SA, Italy; gvigliotta@unisa.it; 5 Dipartimento di Fisica “E.R. Caianiello”, Università degli Studi di Salerno, Via Giovanni Paolo II, 132, 84084 Fisciano, SA, Italy; m.sublimi@sa.infn.it (M.S.S.); fbobba@unisa.it (F.B.); 6 Consiglio Nazionale delle Ricerche, Istituto per la Microelettronica e i Microsistemi (CNR-IMM), Via P. Gobetti 101, 40129 Bologna, Italy; liscio@bo.imm.cnr.it

**Keywords:** star copolymers, films, morphology, mechanical properties, AFM, force volume maps

## Abstract

Star copolymer films were produced by using spin-coating, drop-casting, and casting deposition techniques, thus obtaining ultrathin and thick films, respectively. The morphology is generally flat, but it becomes substrate-dependent for ultrathin films where the planarization effect of films is not efficient. The indentation hardness of films was investigated by Force Volume Maps in both the air and liquid. In the air, ultrathin films are in the substrate-dominated zone and, thus, the elastic modulus *E* is overestimated, while *E* reaches its bulk value for drop-casted ultrathin and thick films. In liquid (water), *E* follows an exponential decay for all films with a minimum soaked time *t*_0_ of 0.37 and 2.65 h for ultrathin and drop-casted ultrathin and thick films, respectively. After this time, *E* saturates to a value on average 92% smaller than that measured in the air due to film swelling. Such results support the role of film morphology in the antimicrobial activity envisaged in the literature, suggesting also an additional role of film hardness.

## 1. Introduction

Branched, star-like copolymers have the ability to form films with physical properties modulated by their topology [1,2,3], albeit those films are usually amorphous or semi-crystalline due to the low degree of crystallinity of star copolymers [2,4]. Among their possible applications, biomedical uses are intensely investigated to date [5]. For instance, the antimicrobial activity of films was recently increased by synthesizing protonable, novel star copolymers [6,7], surpassing the drop in the capacity to kill bacteria over time obtained with other antimicrobial strategies [8,9]. In this case, star-like structures, compared to the linear one, showed higher antimicrobial activity and the best results were obtained with a two-branch copolymer, namely m-PEG-P(MMA-ran-DMAEMA)_2_, insoluble in water and containing ≈40% in mol of non-quaternized 2-(dimethylamino)ethyl methacrylate (DMAEMA), from which films were prepared. Such efficient antimicrobial activity was mainly attributed to enhanced protonation of DMAEMA pendants sustained by the dimerization of vicinal ammonium/amino groups [6,7,10,11,12]. In fact, due to the somewhat limited conformational freedom in the linear structure, mainly DMAEMA groups that are first, or second neighbors, can form the dimers (−N(CH_3_)_2_−H^+^---N(CH_3_)_2_−), while in a star-like structure, the formation of dimers can occur both intra- and inter-chain; the latter within the same macromolecules due to the vicinity of the branches in the region close to the junction (see the molecular structure in Supplementary Materials). Experimentally, star-like structures showed a higher density of surface charges with respect to linear ones, as indirectly confirmed by the measurement of the interaction between polymeric films and benzoate, being the charge density responsible for antimicrobial efficiency [6].

The authors also suggest a possible morphological explanation for the increased charge density, ascribable to the presence of a cavity or channel due to the star-like structure that, allowing the infiltration of water, favored the protonation of the DMAEMA amino pendant groups. The latter idea was supported by the water uptake data and by the glass transition temperature (*T_g_*) values measured after sorption of water, indicating a less compactness of the two-branch structure [6]. Since most of the inherently antimicrobial polymers are cationic and contact-killing, and they do not induce serious microbial drug resistance as they produce physical damage to bacterial cells [12,13], morphological changes induced might be of importance for the rational design of novel antimicrobial, non-inducing resistance materials.

In this scenario, film morphology is pivotal. This work aims to investigate the morphology of m-PEG-P(MMA-ran-DMAEMA)_2_ films in both the air and water, i.e., an environment similar to those used in antimicrobial tests. When immersed in water, polymer films experience swelling [14] and their morphological and mechanical properties change [15]. In accordance, the morphology is statistically investigated in plane and by one-dimensional analysis, while the mechanics are investigated in terms of indentation hardness, measuring the Young modulus and the viscoelastic behavior of films [16,17]. Since the equilibrium swelling is not influenced by film thickness [18], m-PEG-P(MMA-ran-DMAEMA)_2_ films are prepared ultrathin on substrates with a different morphology. The experimental results are always compared to thick films as prepared in Refs. [6,7].

## 2. Materials and Methods

### 2.1. Polymeric Materials

The star copolymer m-PEG-(MMA-ran-DMAEMA)_n_ with *n* = 2 was synthesized at 70 °C in toluene [6]. A 50 mL glass flask was charged, under nitrogen atmosphere, with 0.1 g of m-PEG-Br_2_ macroinitiator and 15 mL of dry toluene. After the dissolution of the macroinitiator, 0.03 g of CuBr, 0.05 g of bpy, 5 mL of MMA, and 2.5 mL of DMAEMA were added. All chemicals used in synthesis process were purchased from Aldrich (St. Louis, MO, USA) and used without any further purification. The mixture, maintained at 70 °C, was magnetically stirred for 18 h and then the reaction was stopped with n-hexane. The copolymer was recovered, dissolved in the minimum amount of chloroform, and passed over a column of activated Al_2_O_3_ to remove the catalyst. The solution was dried in vacuum; the copolymer was recovered, washed with cold methanol, and then dried in vacuum. As measured by Gel Permeation Chromatography (Waters S.p.A., Milano, Italy), the copolymer molecular weight, *M_n_*, and its polydispersity index, PDI, were 89 kDa and 1.4, respectively.

From now on, m-PEG-P(MMA-ran-DMAEMA)_2_ is briefly termed A(BC)_2_ where the block A, m-PEG, is bound to the two blocks BC composed of methylmethacrylate (MMA) and non-quaternized 2-(dimethylamino)ethyl methacrylate (DMAEMA).

The A(BC)_2_ powder was dissolved in chloroform (CHCl_3_) in order to obtain two solutions with concentrations *c* of 1 and 4 mg·mL^−1^ and polymer mass fraction wt.% of 0.067 and 0.268, respectively (see Supplementary Materials).

### 2.2. Silicon Substrates

Substrates were ≈1 × 1 cm^2^ chips prepared by cleaving manually a Si (111) wafer coated with native SiO_x_ (*p*-type, *ρ* = 10 Ω·cm) [19]. Both sides of the Si wafer were mechanically polished (MP), obtaining optically specular surfaces. Finally, one side was finished by chemical mechanical polishing (CMP), obtaining an atomically flat defect-free surface [20]. Before their use, substrates were cleaned by acetone vapors to remove possible physical/chemical contaminants.

### 2.3. Preparation of Ultrathin Polymeric Films by Spin-Coating

Ultrathin polymeric films were prepared by spin-coating 300 µL of A(BC)_2_ solution (*c* = 1 mg·mL^−1^) on silicon substrates. The spin process consists of two steps: (i) The solution was deposited on the substrate by using a mechanical air-cushion pipette (100–1000 µL, Eppendorf Research, Stevenage, UK) placed near the substrate surface (≈2 cm). In order to obtain a homogeneous fluid film on the substrate surface, the solution was deposited after the acceleration stage of the spin-coater when the final rotational speed *ω* was reached (specifically, *ω* is 3000, 3200, 3500, 3750, and 4000 rpm) [21]. (ii) The fluid film was gradually thinned up to its final thickness *h_w_* by keeping the rotational speed *ω* for additional 10 s.

Spin-coated samples were closed within plastic Petri dishes and placed under a chemical hood for 20 h at room temperature to evaporate completely CHCl_3_ from the fluid film. Once dried, ultrathin films are solid, transparent, and insoluble (in water) [22].

The final solid thickness *h_f_* increases with *c* and decreases with *ω*, depending also on the adopted solvent [23]. Since *c* was assumed constant during the spin-coating process, *h_f_* can be evaluated by using Meyerhofer’s equation [24]:

(1) $$h_{f}=wt\%\cdot h_{w}=wt\%{\cdot \left[\left(\frac{3\eta_{0}}{2\rho }\right)\cdot \frac{k}{\left(1-wt\%\right)}\right]}^{1/3}\cdot \omega^{-2/3},$$

where *k* is the mass transfer coefficient, i.e., the amount of polymer transferred from the solution to the substrate [25], *η*_0_ (in cgs, cP = 10^−2^ g·cm^−1^·s^−1^) is the solution viscosity, and *ρ* (in g·cm^−3^) is its density.

The high rotational speeds adopted herein (*ω* ≥ 3000 rpm) should produce ultrathin films with *h_f_* almost independent of *ω* [26]. To confirm this, additional physical parameters characterizing the A(BC)_2_ solution are necessary to calculate *h_f_* from Equation (1) (see Table 1). Since *η*_0_ and *ρ* are unknown for A(BC)_2_, they are assumed equal to those obtained for the polymer MEH-PV, which has similar *M_n_*, 86 kDa, and PDI, 1.52 (MEH-PV is also dissolved in CHCl_3_) [26]. Specifically, the A(BC)_2_ solution has *ρ* = 0.99 g·cm^−3^ and *η*_0_ ≈ 0.61 cP = 0.61 × 10^−2^ g·cm^−1^·s^−1^ for *wt.%* = 0.067 (see Supplementary Materials). The first term in the cube root of Equation (1), i.e., 3*η*_0_/2*ρ*, is ≈0.92 × 10^−2^ cm^2^·s^−1^, while the second term *k* is ≈1.25 × 10^−9^ cm·s^−½^ (calculated from Equation (8) of Ref. [27] with data from Table 1), so the cube root of Equation (1) is ≈ 2.3 × 10^−4^ cm·s^−½^. Accordingly, *h_f_* ranges from ≈11 to ≈9 nm for *ω* = 3000 and 4000 rpm, respectively (rpm was expressed in Hz for dimensional analysis by using the equivalence 1 rpm = 1/60 Hz). These calculated thicknesses confirm the slight dependence of *h_f_* vs. *ω* for high rotational speeds. 

### 2.4. Preparation of Ultrathin and Thick Polymer Films by Drop-Casting and Casting

Ultrathin and thick polymeric films were prepared by drop-casting [30] and casting [22], respectively. Ultrathin films drop-casted, ≈20 nm thick, were obtained by depositing 1 mL of solution on a TEM grid (mesh 300) placed on the CMP substrate. Prepared samples were placed within a Teflon Petri dish under a chemical hood for 24 h so as to evaporate completely CHCl_3_ at room temperature. Once dried, the TEM grid was gently removed obtaining a polymeric solid film composed of square polymeric regions spaced out by 30 µm regions exposing the CMP substrate. In order to measure the thickness of ultrathin films, cross-section profiles across these two regions were performed on topographic images obtained by atomic force microscopy (AFM). The thickness measured by AFM was (16 ± 3) nm (see Supplementary Materials). Thick films, ≈400 μm thick, were self-standing films prepared by dissolving 200 mg of copolymer in 50 mL of CHCl_3_ at room temperature, i.e., *c* = 4 mg·mL^−1^. The solution was cast in a Teflon Petri dish (diameter 3 cm), and the solvent was evaporated at room temperature. The film was removed from the Petri dish and stored in a vacuum oven at 30 °C for three days [7]. Self-standing polymeric films were prepared in the same way reported in the literature [12] and used as reference sample. The average thickness of self-standing films was measured by calipers as (400 ± 25) µm.

### 2.5. X-ray Reflectivity Measurements

X-ray reflectivity (XRR) measurements were performed using a SmartLab-Rigaku (Assing S.P.A., Roma, Italy) diffractometer equipped with a rotating anode (Cu Kα, λ = 1.54180 Å), followed by a parabolic mirror to collimate the incident beam and a series of variable slits (placed before and after the sample position) to reach an acceptance of 0.01°.

### 2.6. Atomic Force Microscopy Imaging

The microscope used for all measurements was a JPK Nanowizard III equipped with Vortex electronics (Bruker Nano GmbH, Berlin, Germany). Polymeric films were first topographically investigated by using the amplitude modulation atomic force microscopy technique (AM-AFM) under ambient conditions. All three available MikroMash NSC35 (Innovative Solutions Bulgaria Ltd., Sofia, Bulgaria) cantilevers with nominal resonant frequency ω_0_ of ≈150, ≈200, and ≈300 kHz and correspondent nominal spring constant *k* of ≈6, ≈9, and ≈16 N·m^−1^ were employed for AM-AFM (when necessary, the real *k* was calculated through the Sader method [31]).

The mechanical properties of the polymeric films were evaluated by Force Volume Maps (FVM) obtained by using the Quantitative Imaging (QI) mode developed by JPK Instruments [32]. In FVM mode, multiple force curves were acquired at points (pixel, px) of a defined grid pattern (in our case, 128 × 128 px^2^). The interactions between the tip and sample were measured locally and mapped point-by-point through force−distance curves. In particular, the tip was moved toward (approach curve) and away from (retraction curve) the sample surface at each point of the grid pattern, while the cantilever deflection (in V) was continually registered with respect to the position of the piezoelectric actuator (in µm, termed “height”) [33]. Prior to measurements, the elastic constant *k* of the cantilever was calibrated in air on bare thermal SiO_2_ substrate by performing a force curve on a single point of the SiO_2_ surface [34]. The slope of the linear part after the jump-to-contact point was reciprocal to the cantilever sensitivity *s* (in nm·V^−1^) and it was used to convert in nm the cantilever deflection measured from the photodiode (in V). Once *s* was measured, *k* was calculated by using the thermal tune method (see Supplementary Materials of Ref. [35]). FVMs were obtained by fixing the maximum applied force *F_max_* (in nN) calculated from Hook’s law *k*·*U*·*s*, where the cantilever deflection *U* (in V) was kept constant at 0.5 V for all measurements [36]. Force curves composing the FVM had a fixed maximum path length of 50 nm, which was traveled in 200 ms (approach and retraction paths). QI experiments were performed in both air (ambient temperature *T_a_* = (27 ± 2) °C and relative humidity RH = (62 ± 5)% [37,38]) and liquid (mQ water). In liquid measurements, the sample was placed in the center of a homemade pool built by fixing a polypropylene tube 5 mm long (outer diameter Ø = 24 mm, inner Ø = 23 mm) on a glass microscope slide. Both the tube and sample were fixed on the glass slide by using the JPK bio-compatible glue [39]. The total volume of the pool was about 1.5 mL, which granted up to 8 h of consecutive measurements (for longer measurements the pool was refilled). QI measurements employed Bruker RTESP, LTESP (Bruker Nano GmbH, Berlin, Germany), and MikroMash NSC35 (Innovative Solutions Bulgaria Ltd., Sofia, Bulgaria) silicon cantilevers with ω_0_ ≈ 180, 190, and ≈290 kHz, respectively, and calibrated elastic constants *k* of ≈35, ≈46, and ≈32 N·m^−1^. 

Topographic images were analyzed with the software Gwyddion (version 2.40) [40], while QI images were analyzed by the JPK Data Processing software (version spm-5.1.8).

## 3. Results and Discussions

### 3.1. Morphological Characterization of Silicon Substrates

**Figure 1:**
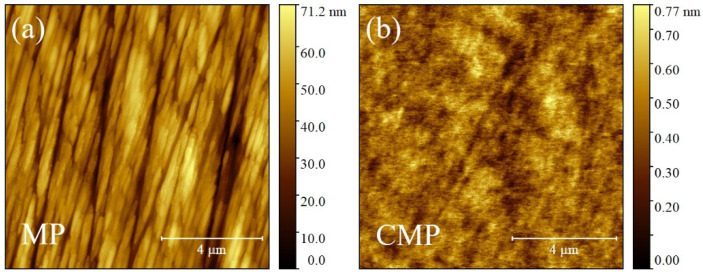
Topographic AFM images 10 × 10 µm^2^ of mechanical ((**a**), MP) and chemical mechanical ((**b**), CMP) polished wafer surfaces. The maximum value of false color map ruler is reduced by two orders of magnitude from MP to CMP, stressing roughness differences.

The morphology of wafer side surfaces, i.e., mechanical polished (MP) and chemical mechanical polished (CMP), are characterized by AM-AFM. The MP surface shows features in the order of tens of nm due to mechanical finishing (see Figure 1a), while the CMP surface is flat with details below a nm (see Figure 1b). Accordingly, the root mean square roughness *R_q_* reduces from (10.7 ± 1.2) to (0.10 ± 0.025) nm for MP and CMP, respectively. 

Such *R_q_* values are comparable to those reported in the literature [41] where CMP wafers were polished with different abrasive SiC papers and velvet rugs imbued with Al_2_O_3_ slurry, assessing the progressive evolution of roughness parameters vs. finer polishing. The *R_q_* value of the MP side is consistent with a surface polished by SiC papers with a grit higher than 400 (possibly 1200 [42]), while the CMP side has an *R_q_* value even lower than the one reported in the literature and defined “not machined surfaces” [41] (due to higher surface cleanliness—compare Figure 1b,d of Ref. [41]).

**Figure 2:**
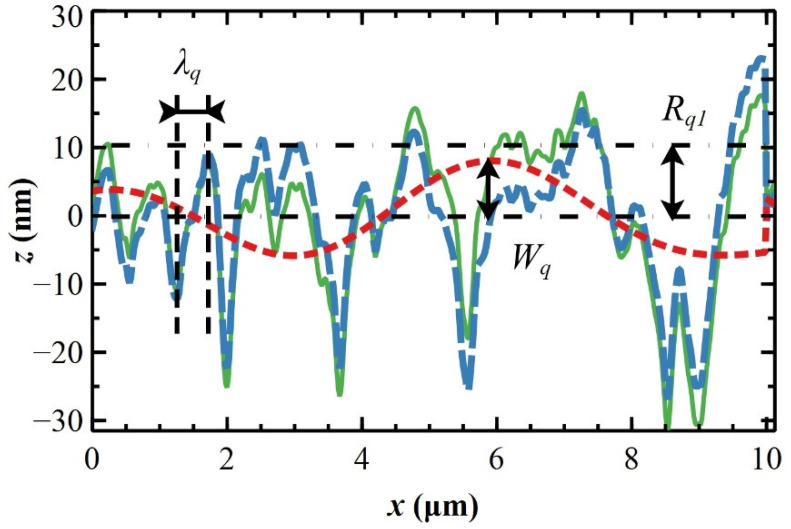
Typical topographic profile in the *z*–*x* plane; *z* is the height variation and x is the direction orthogonal to polishing features, obtained by averaging 90 adjacent profiles from MP AFM images (continuous green line). One-dimensional analysis splits the profile into waviness (red dashed line) and roughness (blue dashed line) profiles that are characterized by an average amplitude *W_a_* and wavelength *λ_q_* and a root mean square roughness *R_q_*_1_, respectively.

To evaluate the roughness parameters of the MP surface, a one-dimensional analysis of averaged topographic profiles is used [40,43]. The average profile is obtained by averaging 90 adjacent profile lines along the direction orthogonal to the polishing features. The one-dimensional analysis splits the averaged topographic profile into waviness (low-frequency components, corresponding to the polynomial background of the image) and roughness (high-frequency components) [44]. Through this analysis, hidden surface oscillations with specific amplitudes and wavelengths emerge even if, in principle, the MP surface should not have them (see Supplementary Materials). The splitting procedure depends critically on the cut-off *C* that, set to 0.0098, correctly splits the MP surface profile (see Figure 2 and C calculations in Supplementary Materials).

The profile of the surface roughness obtained by one-dimensional analysis on 10 × 10 µm^2^ topographic images (blue dashed line in Figure 2) has a roughness *R_q_*_1_ of (8.5 ± 0.1) nm and a root mean square wavelength *λ_q_*, i.e., the average peak-to-valley distance [45], of (0.72 ± 0.03) µm. The waviness is characterized by a root mean square amplitude *W_q_* of (1.8 ± 0.6) nm (red dashed line in Figure 2). The sum of *W_q_* and *R_q_*_1_ is (10.3 ± 0.7) nm, which is equal, within experimental error, to the roughness *R_q_* calculated from height distribution [46].

### 3.2. X-ray Characterization of Ultrathin Polymeric Films

**Figure 3:**
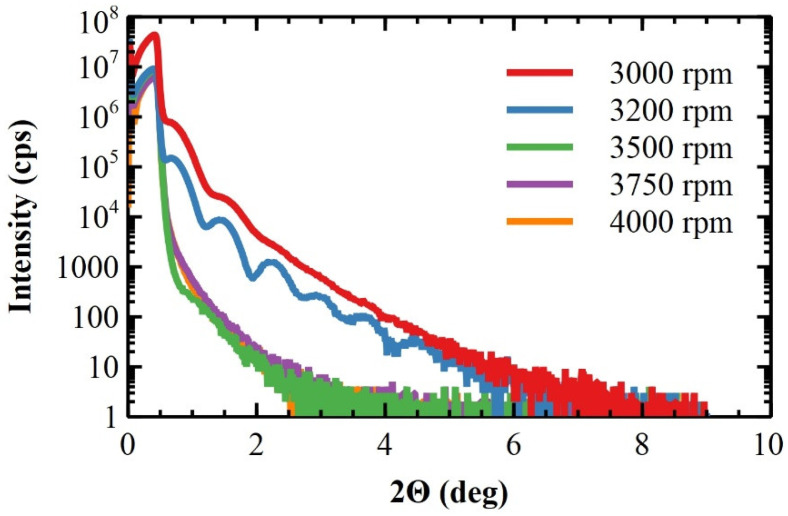
XRR scans of ultrathin polymeric films deposited on CMP and MP substrates at 3000, 3200, 3500, 3750, and 4000 rpm, respectively.

Two distinct behaviors are clearly visible in the XRR curves on the MP and CMP substrates (see Figure 3). The polymeric films deposited on the CMP substrates, at ω = 3000 and 3200 rpm, exhibit Kiessig fringes generated by the constructive interference of the reflected X-ray beam by both the polymeric film surface and the film/substrate interface [47]. Such fringes are indicative of a smooth film surface and a smooth film/substrate interface, in agreement with data obtained by AFM. The thickness of the polymeric films can be measured from Kiessig periodicity, obtaining a thickness range of [10,12] nm for both films [48,49]. Notably, such values are similar to those theoretically calculated in Section 2.3. The reduction in Kiessig amplitude intensity for the film deposited at ω = 3000 rpm indicates an increased roughness of the film surface with respect to that deposited at ω = 3200 rpm, in line with the smoothing effect expected for increasing ω on films deposited on CMP substrates. Conversely, the XRR curves of polymeric films deposited on MP substrates at ω ranging from 3500 to 4000 rpm do not show Kiessig fringes, and the XRR signal is damped. This is typical of ultrathin films deposited on (relatively) rough surfaces [50], as observed by AFM.

### 3.3. Morphological Characterization of Ultrathin and Self-Standing Polymeric Films

**Figure 4:**
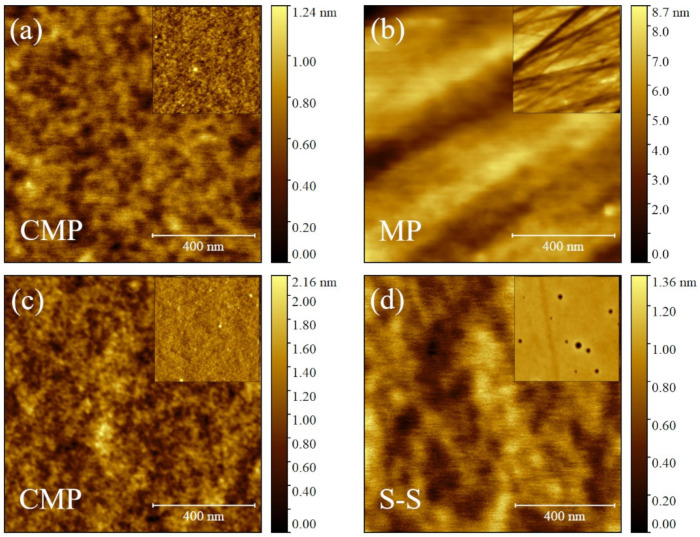
Topographic AFM images of polymeric films deposited by spin-coating on CMP (**a**) and MP (**b**) substrates at 3200 and 4000 rpm, respectively; drop-casting on CMP substrates (**c**); self-standing films, S-S, obtained by casting (**d**). All images are 1 × 1 µm^2^. Insets: topographic images at larger scale (3.5 × 3.5 µm^2^) to show morphological features of films (**a**) or their flatness (**b**–**d**).

The morphology of polymeric films depends strongly on the deposition technique. In spin-coated films, it depends on both the substrate, CMP or MP, and the rotational speed ω. This latter dependence is lost in the case of CMP substrates where the films are flat with an average roughness *Rq* of ≈0.17 nm (see Table 2) and the films are featureless even at a large scale (see Figure 4a and its inset).

Otherwise, the film morphology depends on *ω* due to the morphology of MP substrates (see Figure 4b). By comparing Figure 1a and Figure 4b, polymeric films smoothen the topographical features of bare MP substrates, although relatively large scratches are still present. The roughness of polymeric films, measured both by one-dimensional analysis, *R_q1_*, as well as height distribution, *R_q_*, are progressively reduced vs. *ω* from *R_q_*_1_ ≈ 4.1 to ≈2.6 nm for 3500 and 4000 rpm, respectively (see Table 2). Such a reduction, but less pronounced, is also observed on *W_q_*. Within experimental errors, *λ_q_* remains, on average, constant at (0.74 ± 0.30) µm and independent of *ω* (see Table 2).

For understanding the morphological evolution of polymeric films vs. *ω*, the one-dimensional parameters summarized in Table 2 are compared with those obtained on bare MP substrates (*λ_q_* ≈ 0.72 µm, *W_q_* ≈ 1.8 nm, and *R_q_*_1_ ≈ 8.5 nm). The wavelength *λ_q_*, which is determined by surface scratches, is unaffected by the presence of films and invariant with *ω*. Since the scratches are deeper, or at most comparable, to the film thickness *h_f_* (on average ≈ 11 nm, cp. to Section 3.2), their modulations are preserved even after the film deposition. The amplitude *W_q_* also depends on the scratches, but it is reduced with respect to the bare MP substrate for films deposited at *ω* = 4000 rpm. The roughness *R_q_*_1_ is lower than the roughness of the bare MP substrate, also for increasing *ω*. Since *R_q_*_1_ is governed by small height variations around the oscillating roughness profile (see Figure 2), its reduction means that such height modulations are progressively filled by the film for increasing rotational speed *ω.*

These experimental observations can be rationalized with a single parameter termed surface planarization *P* (in %) [51]. Mathematically, *P* is defined as

(2) $$P=100\left[1-\frac{s_{h}}{d}\right]$$

where *s_h_* and *d* are the (average) peak-to-valley roughness, viz., *R_z_* (ISO) [52], for the polymeric film and the bare MP substrate, respectively. If the film is conformal to the substrate features, *s_h_* → *d* and *P* → 0%. Vice versa, if the film is flat *s_h_* → 0 and *P* → 100%. For bare MP substrates, *d* is (35 ± 6) nm and *s_h_* ranges from ≈18 to ≈9 nm for 3500 and 4000 rpm, respectively (see Table 2). Accordingly, *P* runs from ≈49 to ≈73%. In the case of spin-coated films on CMP substrates, they are flat with a small *d* of (0.15 ± 0.05) Å. The same for *s_h_*, that is, ≈0.15 nm and ≈0.2 nm for 3000 and 3200 rpm, respectively (see Table 2). Substrates and films have comparable *R_z_* (ISO), so films are flat (*P* = 100%), as well as the CMP substrate.

Polymeric films on CMP substrates deposited by drop-casting are flat and featureless with an *R_q_* of (0.25 ± 0.025) nm, even at a large scale size (see Figure 4c and its inset). Similarly, self-standing polymeric films S-S have a surface roughness *R_q_* of (0.20 ± 0.025) nm (see Figure 4c and its inset).

In view of the *P* values reported in Table 2, MP substrate planarization through the polymeric film is affected by both substrate corrugations and *ω*. Such nanometer corrugations are also expected to locally change the film thickness.

The fluid film formed on the substrate surface during the spin-coating deposition is pivotal for the planarization effect. The A(BC)_2_ solution is a non-Newtonian fluid due to the high volatility of CHCl_3_ and, also, it is a low viscosity fluid due to the low concentration of the solution (*c* = 1 mg·mL^−1^) and the low polymer mass fraction dissolved in CHCl_3_ (*wt.*% = 0.067). Such a fluid easily fills completely the scratches independently of *ω* [53]. Such filling is also facilitated by the average width of the scratches (a few hundred nm); indeed, the lowest critical width at which trench filling is impeded is about 5 cm (as calculated from the spin-coating theory in our experimental conditions [54]), several orders of magnitude larger than the average width of the scratches. Accordingly, the liquid film spin-coated on the substrate fills the scratches completely and, after solvent evaporation, surface planarization is reached even if it is not perfect due to the non-Newtonian behavior of the solution [54].

**Figure 5:**
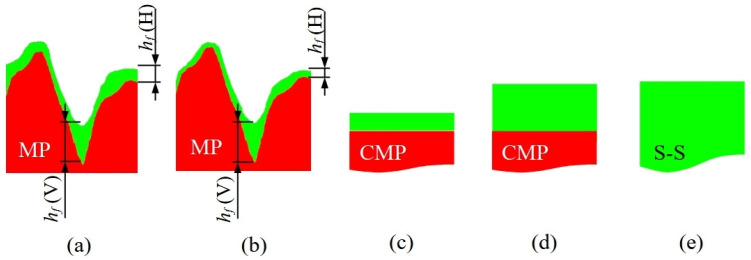
Sketches of solid films (green regions) cross-sections obtained by spin-coating on MP substrates (red regions) at *ω* = 3500 rpm (**a**) and 4000 rpm; (**c**) spin-coating on CMP substrates (**c**); (**d**) drop-casting on CMP substrates; (**e**) casting on Teflon Petri dish (self-standing film—S-S, cp. to Section 2.4). Polymeric films on MP substrates present several details on valleys, *h_f_* (V), and hills, *h_f_* (H), due to wetting that modulates locally the final film thickness *h_f_*. On flat regions, *h_f_* is thicker for low *ω* (**a**) and thinner for high ones (**b**), while in valleys it remains constant.

The (relatively) flat regions between the scratches show a different behavior. The liquid film thickness *h_w_* is thinned for increasing *ω* similarly to flat substrates like CMP. The solid film thickness *h_f_* is reduced from 10 to 9 nm passing from *ω* = 3500 to 4000 rpm (cp. to Section 2.3), a thickness comparable to small height variations determining *R_q_*_1_ (≈8.5 nm). In these conditions, height variations are smoothed by the film [55,56] and *R_q_*_1_ is reduced from ≈ 8.5 nm (bare substrate) to ≈4.1 nm (or less; see Table 2). The liquid film spin-coated on the substrate is governed by capillary forces (Ω^2^ ≈ 10^−7^ [57]), and the solution moves toward roughness valleys (≈100 nm wide, as evaluated by the Height–Height Correlation Function [58]) rather than on top of hills due to their high aspect ratio [59]. Since *h_f_* is larger for a lower *ω*, the dried film on roughness hills, *h_f_* (H), will be thicker at 3500 to 4000 rpm while the roughness valleys, like scratches, will be filled completely by the solution and *h_f_* (V) will be independent to *ω* (see Figure 5a,b). Consequently, *h_f_* (H) obtained at ω1, *h_f_* (H)|_ω1_, is thicker than *h_f_* (H)|_ω2_ if ω1 < ω2 while *h_f_* (V)|_ω1_ = *h_f_* (V)|_ω2_ regardless of ω. As reported by Table 2, *R_q_*_1_|_ω1_ > *R_q1_*|_ω2_ for ω1 < ω2, explaining why *P*|_ω1_ < *P*|_ω2_. These observations and results suggest that polymeric films on MP substrates have a final thickness *h_f_* comparable to the substrate roughness in agreement with X-ray results and the literature [60]. Other films on the CMP substrate (Figure 5c,d) and self-standing (S-S, Figure 5e) are featureless and do not need additional morphological descriptions.

### 3.4. Elastic Modulus of Polymeric Films Measured in the Air

**Figure 6:**
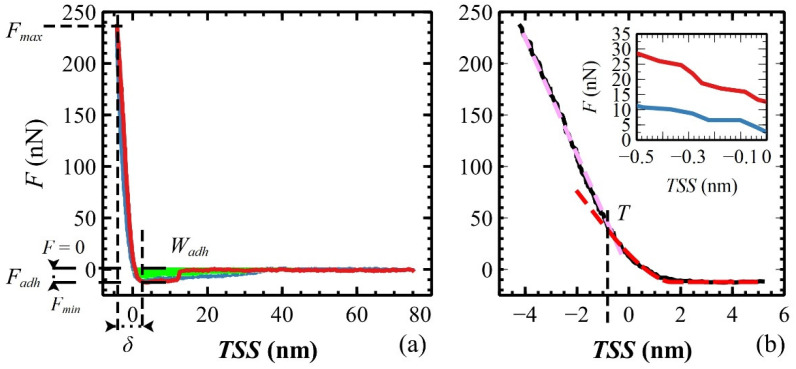
(**a**) Typical force *F* vs. Tip Sample Separation (*TSS*, approach curve—red, retraction curve—blue) obtained on polymeric films with all measurable parameters: *F_max_*, *F_adh_*, *δ*, and *W_adh_* (green area); (**b**) zoom of the approach curve (black) in the tip–sample contact region. In the first few nm, from 5 to −2 nm, the sample is elastically deformed by the tip and Hertz model fits properly the curve (red dashed line). Then, the sample begins to be plastically deformed at point *T*, where the curve changes its slope (pink dashed line). Inset: zoom of *F* vs. *TSS* curve near *TSS* = 0 (approach curve—red, retraction curve—blue) for highlighting the small curve hysteresis symptomatic of elastoplastic behavior of the film.

The indentation hardness of polymeric films was measured by FVM [61,62]. Raw force–height curves composing the FVM were vertically aligned to the *x*-axis (baseline subtraction, *y* = 0) and horizontally shifted to the *y*-axis by setting the height value to *x* = 0 at *F* = 0, i.e., where the tip–sample interaction starts to be in a repulsive regime (also termed “contact point”; the measured height is, therefore, re-scaled). To perform quantitative measurements of the mechanical properties, force–height curves have to be converted into force–tip–sample separation (*TSS*) curves [63] by subtracting the bending of the cantilever from the height (see Figure 6a). As reported by Cappella [34], the approach curve is used to measure the indentation *δ* and elastic modulus *E*, while the retraction curve is used to measure the adhesion force *F_adh_*, and the work required to separate the tip from the sample, i.e., the work of adhesion *W_adh_* (see Figure 6a). Such physical parameters for each sample were obtained by analyzing one hundred *TSS* curves, manually selected on random spatial positions from FVM composed of 128 × 128 curves. Then, the data were plotted as histograms and fitted by Gaussian distributions to obtain the average value of the parameters.

To measure *E* of the polymeric films, the Hertz model was adopted wherein the tip is approximated to a sphere [64]. This approximation is valid for a tip indentation *δ* smaller than the radius of the curvature of the tip *Ξ*. In these experiments, *Ξ* = 10 nm is calculated by averaging the maximum nominal *Ξ* reported in the datasheets (12, 12, and 8 nm for Bruker RTESP, LTESP, and MikroMash NSC35, respectively), so *δ* < *Ξ* for all measurements (Table 3). The choice of the Hertz model is also validated by the adhesion force *F_adh_* measured as the difference between the minimum force, *F_min_*, and the baseline, i.e., *F* = 0 (see Figure 6a) [65,66]. Within the Hertz model, the calculation of *E* is precise if the maximum applied force *F_max_* is much larger than *F_adh_* [67,68,69]. In our films, *F_adh_* runs from a minimum of ≈ 6 nN (CMP-DC sample) to a maximum of ≈19 nN (CMP-SC sample) with *F_max_* ≈ 120 nN and ≈260 nN, respectively (see Table 3). The ratio *F_adh_*/*F_max_* is within the range [0.05, 0.07], hence *E* is measured correctly.

The inset of Figure 6b shows a small hysteresis between the approach and retraction curves, typical of an elastoplastic deformation of the films [34]. The indentation *δ*, measured as the difference between the *TSS* values at *F_min_* and *F_max_* (see Figure 6a) [71], shows two slopes indicated by two dashed lines in Figure 6b. The portion of the approach curve from the *F_min_* plateau to the intersection *T* is the elastic deformation of the film, useful for measuring *E* (the first few nm; the red dashed line is the fitting curve obtained by using the Hertz model). In this indentation range, *F* increases from 0 to ≈30 nN with a root mean square error of (1.0 ± 0.2) nN, i.e., the quality of data fitting is within an error of 3%. Then, the film is plastically deformed by the tip for an additional few nm, as indicated by the pink dashed line (a guide to the eye) [72]. The last parameter adopted in the Hertz model is the Poisson ratio of polymeric films ν, fixed to 0.33 from the literature [15]. This choice is supported by experimental results on similar amorphous polymeric films (see SI of Ref. [15]), where the magnitude of *E* shows slight changes within a realistic ν interval, i.e., 0 < ν < 0.5 and, also, the trend of *E* vs. film thickness *h_f_* is preserved for all ν.

**Figure 7:**
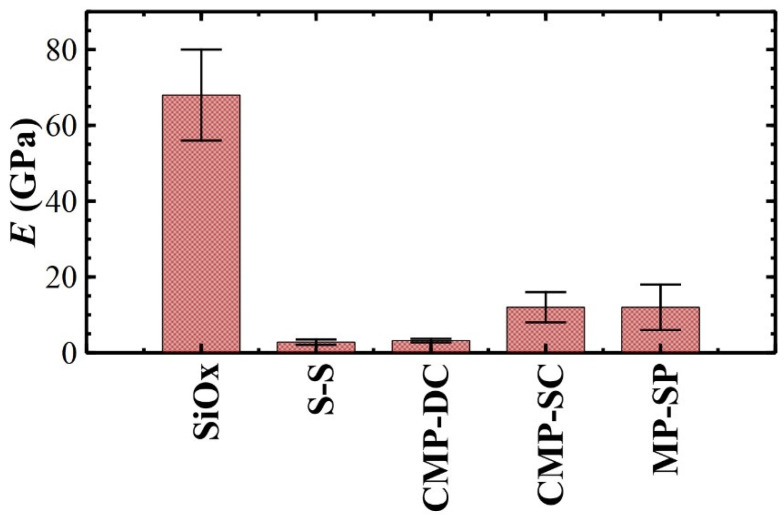
Young moduli *E* of polymeric films deposited by casting, drop-casting (DC), and spin-coating (SC) on CMP and MP substrates, except for self-standing films (S-S). Such values are compared to *E* of SiO_x_ [75].

As shown in Figure 7, thicker films have comparable *E* (≈3 GPa; see Table 3), in agreement with the results obtained on similar bulk films (1–10 µm thick) [73]. Our polymeric films reach the bulk value for *h_f_* ≥ 20 nm, independently of *c* (S-S and CMP-DC were obtained from solutions with *c* = 4 and 1 mg·ml^−1^, respectively) and in agreement with the literature [15,74]. For ultrathin films deposited by spin-coating, *E* increases to ≈12 GPa with a variance of ≈0.3 and ≈7 for CMP-SC and MP-SC, respectively. Such high variance produces *E* values spanning from ≈7 to ≈20 GPa for MP-SC samples, suggesting that *h_f_* is locally not homogeneous by reason of the high local roughness *R_q_*_1_ with respect to the flatness of CMP substrates.

The film thickness *h_f_* plays a key role in the *E* interpretation; *E* is accurate if the ratio *δ*/*h_f_* is ≤0.025 (film-affected zone); otherwise, it is overestimated if *δ*/*h_f_* > 0.15 (substrate-dominated zone) [66].

The S-S sample is the reference for the film-affected zone: a tip indentation *δ* of ≈6 nm, obtained by applying a maximum force *F_max_* of ≈240 nN (see Table 3) on a sample ≈ 400 µm thick, produces a *δ*/*h_f_* of ≈1.5 × 10^−5^. On CMP-DC, i.e., the thickest ultrathin film, *δ* is ≈ 3 nm, obtained by applying a *F_max_* of ≈ 120 nN (see Table 3) on a ≈20 nm thick film, i.e., *δ*/*h_f_* ≈ 0.15, a value between film- and substrate-dominated zones (transition zone). In particular, *δ*/*h_f_* ≈ 0.15 is the upper limit to avoid the substrate effect, explaining why the measured *E* is comparable to the S-S sample. In the case of the ultrathin films prepared by spin-coating on the CMP and MP substrates, *E* was measured in the flat regions between the MP substrate scratches, which are morphologically similar to the flat CMP substrates (but with higher roughness). The CMP-SC films are flat with a constant thickness *h_f_* ≈ 12 nm; *δ* is ≈2.6 nm by applying *F_max_* of ≈260 nN, so *δ*/*h_f_* ≈ 0.22 > 0.15 and *E* is overestimated (substrate-dominated zone). In the MP-SC samples, the ultrathin films are thinner by 1 nm (at least) than those prepared on the CMP substrates because of a higher rotational speed during deposition (*ω* = 3500 rpm compared to *ω* = 3200 rpm). The thickness *h_f_* is <11 nm, *δ* is ≈1.6 nm for *F_max_* ≈ 120 nN, and *δ*/*h_f_* > 0.15 (at the minimum), so just enough to enter the substrate-dominated zone. Accordingly, the CMP-SC and MP-SC samples have comparable *E*. Such an overestimation of *E* on ultrathin films is reported in the literature [73,76,77,78], and it is associated with both a supporting substrate [76] and polymer molecular weight [79]. In addition, it can also be explained by using an extreme case study termed “contact-induced stiffening” [80], i.e., when the substrate is elastically deformed by the tip after a full plastic deformation of the film [66].

The adhesion between the tip and the sample increases for an increasing interaction time, i.e., for increasing indentation *δ* [81]. This phenomenon is due to the increase in the effective surface area of the tip interacting with the sample, resulting in an increase in the overall adhesion between the tip and the sample. During sample indentation (approach curve), the tip interacts with the sample by van der Waals forces and H-bond [82]. The sum of such interactions increases for an increasing effective surface area; therefore, the adhesion force *F_adh_* is expected to increase with an increase in the maximum applied force *F_max_* (see Table 3) [81]. Once indentation is complete, the tip is retracted from the surface (retraction curve) and the work to detach the tip from the sample (adhesion work, in J) is the work necessary to break the van der Waals forces and H-bond (material-dependent), and then to overcome capillary forces [83]. As shown in Table 3, *F_adh_* and *W_adh_* depend on film thickness with higher values for ultrathin films made by spin-coating since their thickness is close to the critical one (≈10 nm) [84]. By comparing the data in Table 3 with the literature, *F_adh_* is comparable to the one obtained on PMMA films [85] suggesting that MMA branches might be exposed at the film surface.

### 3.5. Elastic Modulus of Polymeric Films Measured in Liquid

**Figure 8:**
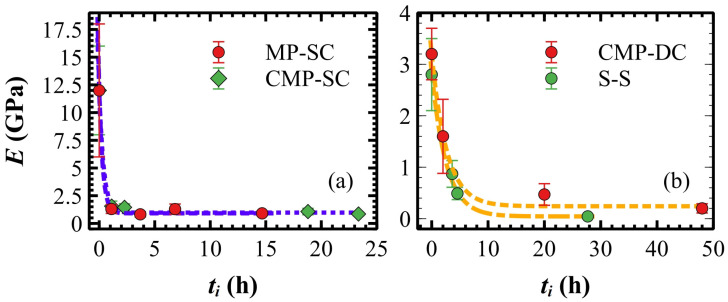
Exponential decay (dashed lines) of *E* vs. *t_i_* for ultrathin (**a**) and drop-casted ultrathin and thick (**b**) films. For convenience, *t_i_* measured in sexagesimal was converted in centesimal.

When polymeric films are immersed in mQ water, a certain amount of water is soaked up into the film over time [86]. Film confinement leads to a decrease in the water diffusion coefficient [87], so ultrathin films are expected to be less permeable to water than thick ones. As shown in Figure 8, *E* decreases exponentially for increasing immersion time *t_i_* with a time constant *t*_0_, defining the minimum soaked time after which the mechanical properties of wet samples saturate to *E_S_* [88]. As expected, *t_0_* and *E_S_* depend on the thickness *h_f_* (see Table 4): (i) ultrathin films show a shorter minimum soaked time *t*_0_ ≈ 0.37 h (Figure 8a) with respect to thick and drop-casted ultrathin films that take more time to soak up water, *t*_0_ ≈ 2.65 h (Figure 8b); (ii) wet samples reduce their elastic modulus by about 92% and notably by 98% for the S-S samples. By comparing ultrathin films deposited on the same substrate, viz., CMP-SC and CMP-DC samples, *E_S_* for the former is about four times larger than the latter (see Table 4) and, thus, the *E* ratio observed in the air is preserved in water (cp. to Table 3).

**Figure 9:**
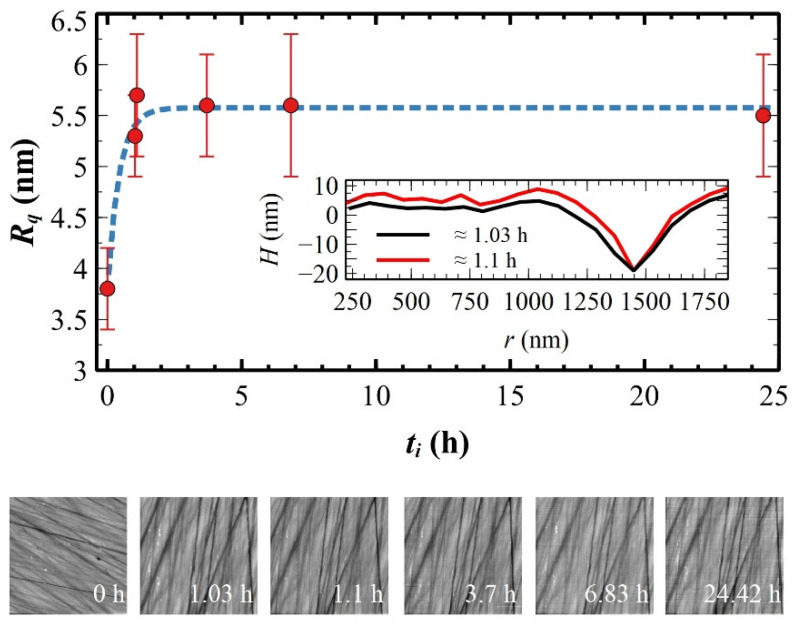
Exponential growth of *R_q_* vs. *t_i_* (dashed blue line). The *R_q_* value at *t_i_* = 0 was measured in air on the same sample but not in situ. For convenience, *t_i_* measured in sexagesimal was converted in centesimal. Inset: cross-sections across a flat region measured in the same position of the surface from two consecutive images (1.03 and 1.1 h). Below: sequence of topographic images collected for increasing *t_i_* in the same position (in situ, except the first one at *t_i_* = 0).

Films on the CMP substrates were used to test how the indentation *δ* changes for a fixed immersion time (*t_i_* ≈ 2 h). In the air, the CMP-SC films show a *δ* of ≈2.6 nm by applying a maximum force *F_max_* of ≈260 nN, whereas, after ≈2 h of immersion, the same indentation (*δ* ≈ 2.8 nm) is obtained with one-fifth of the force (*F_max_* ≈ 45 nN). On the CMP-DC films, *δ* is doubled after *t_i_* ≈ 2 h, increasing from ≈5.8 nm (in the air) to ≈10.6 nm (in water) by applying half of the force (240 vs. 110 nN). Such mechanical behavior is caused by the film swelling [14]. On featureless surfaces, like the CMP-SC and CMP-DC samples, the swelling is observable only by X-ray or ellipsometry measurements [14,89], whereas surfaces rich in morphological features is necessary for AFM measurements [90]. This is the case of MP-SC samples that are characterized by flat regions between deep scratches (cp. to Section 3.3) where swelling depends on the substrate morphology: it is large in flat regions and small within scratches due to the confinement effect of the scratch walls [91,92]. Such local film expansions produce an increase in the surface roughness *R_q_* for all immersion times [92,93]. The roughness grows and saturates following an exponential saturating curve characterized by a time constant *t_R_* = (0.44 ± 0.16) (Figure 9, blue dashed line). Thanks to in situ FVMs, the same topographic profile crossing a deep scratch (>20 nm, taken as the reference) was collected in two consecutive FVMs at *t_i_* ≈ 1.03 and 1.1 h, i.e., where *R_q_* starts to saturate. As shown in the inset of Figure 9, film swelling in the flat region is clearly visible. In situ FVMs performed in liquid also confirm that the film is water-insoluble (see sequence of images in Figure 9) [7]. As expected in liquid [83], *F_adh_* and *W_adh_* are largely reduced and constant for all the samples within experimental errors (see Table 4), confirming that capillary forces give the main contribution to the tip–sample adhesion.

In view of these results, the morphological interpretation reported in the introduction and envisaged in Ref. [6] appears to be correct. The swelling of the star copolymer network causes a stretching of the A, B, and C components. Depending on the cross-link density, the network architecture, and the polymer–solvent interaction, the swelling equilibrium is reached at different amounts of solvent uptake [92], making the film softer at the surface. Notably, the minimum soaked time *t*_0_ for thick films (2.65 h) includes 1.5 h for having high effectiveness of antimicrobial activity due to charges [7] and the stress time of bacteria membranes [12]. Lastly, the large reduction in *E* for the S-S samples might promote a conformal contact between the bacteria and film, thus enhancing all chemical/physical phenomena related to antimicrobial activity.

## 4. Conclusions

Star copolymer films were produced by spin-coating, drop-casting, and casting deposition techniques obtaining ultrathin and thick films, respectively. Drop-casted ultrathin and thick films were morphologically flat, while spin-coated ultrathin films had a morphology dependent on the substrate. In the case of a rough substrate, polymer films smoothed the substrate surface except for (relatively) deep scratches. The indentation hardness of such films was investigated by FVMs in both the air and liquid. In the air, ultrathin films were in the substrate-dominated zone and, thus, the elastic modulus *E* was overestimated, while *E* reached its bulk value for drop-casted ultrathin and thick films, specifically for thicknesses > 20 nm. The surface adhesion was correlated to the film thickness showing larger adhesion in ultrathin films with respect to drop-casted ultrathin and thick films. In liquid (water), *E* followed an exponential decay for all films with a minimum soaked time *t_0_* of 0.37 and 2.65 h for ultrathin and drop-casted ultrathin and thick films, respectively. After this time, *E* saturated to a value that was reduced by about 92% or more for all films. Such film softening was caused by swelling. These results are consistent with the morphological picture envisaged in Ref. [6] and, moreover, suggest a role of mechanical properties in the antimicrobial activity. Since *t_0_* is short with respect to the timescale of standard FVMs, Fast Force Mapping Mode [94] is indispensable for investigating the first two hours of the swelling process, which are crucial for understanding changes in film hardness.

## Figures and Tables

**Table 1:** Physical parameters of the A(BC)_2_ solution useful to calculate *k* from Equation (8) of Ref. [27].

A(BC)_2_ Data
*T* = 298.15 K
*R* = 82.06 atm·cm^3^·mol^−1^·K^−1^
*D_g_* = 0.106 × 10^−6^ cm^2^·s^−1 a^
*ν_g_* = 0.1553 cSt = 0.1553 × 10^−2^ cm^2^·s^−1 b^
*P_CHCl3_* = 26.271 kPa ≈ 0.26 atm ^c^
*M_CHCl3_* = 119.38 g·mol^−1 d^
*ρ*_0_ = 1.49 g·cm^−3 e^
*C* = 0.5474 ^f^

^a^ *D_g_* is the binary diffusivity of the solvent in the overhead gas phase [28]; ^b^ *ν_g_* is the kinematic viscosity of the overlying gas [28]; ^c^ *P_CHCl3_* is the vapor pressure of pure chloroform (CHCl_3_) at temperature *T* [29]; ^d^ *M_CHCl3_* is the molecular weight of chloroform; ^e^ *ρ*_0_ is the density of pure chloroform; ^f^ *C* depends on the Schmidt number of the overlying gas [27,28].

**Table 2:** Surface roughness parameters obtained by one-dimensional analysis of polymeric films deposited on MP and CMP substrates at increasing rotational speed ω. Average roughness *R_q_*, planarization *P*, and peak-to-valley roughness *s_h_* are related to the whole surface, while root mean square wavelength *λ_q_*, average amplitude *W_q_*, and root mean square roughness *R_q1_* were obtained by one-dimensional analysis of averaged topographic profiles.

Ω (rpm)	Sub	*R_q_* (nm)	*W_q_* (nm)	*λ_q_* (µm)	*R_q1_* (nm)	*s_h_* (nm)	*P* (%)
3000	CMP	0.15 ± 0.025	/	/	/	≈0.14	≈100
3200	CMP	0.19 ± 0.125	/	/	/	≈0.19	≈100
3500	MP	6.4 ± 0.4	2.5 ± 1.9	0.60 ± 0.15	4.1 ± 0.8	18 ± 3	49 ± 17
3750	MP	4.5 ± 0.9	2.1 ± 0.7	0.98 ± 0.36	3.0 ± 1.3	13 ± 3	63 ± 25
4000	MP	4.0 ± 0.5	1.2 ± 0.2	0.65 ± 0.29	2.6 ± 0.4	9.3 ± 0.1	73 ± 13

**Table 3:** Sample properties extracted from *F*-*TSS* curves analysis: tip indentation *δ*, maximum force exercises by the tip *F_max_*, adhesion force *F_adh_*, and work of separation *W_sep_* [70].

Sample	*δ* (nm)	*F_max_* (nN)	*F_adh_* (nN)	*W_sep_* (×10^−17^ J)	*E* (GPa)
MP-SC	1.6 ± 0.3	121 ± 5	14 ± 1	6.8 ± 0.7	12 ± 6
CMP-SC	2.6 ± 0.5	257 ± 4	16.8 ± 1.6	8 ± 1	12 ± 4
CMP-DC	4.2 ± 0.3	120.4 ± 0.6	7.7 ± 0.1	3.6 ± 0.8	3.2 ± 0.5
S-S	5.8 ± 0.5	240 ± 1	8.2 ± 1.6	3.3 ± 0.6	2.8 ± 0.7

**Table 4:** Mechanical properties of films immersed in water.

Sample	*t_0_* (h)	*E_S_* (Gpa)	*F_adh_* (nN)	*W_adh_* (×10^−17^ J)
MP-SC	0.33 ± 0.08	0.91 ± 0.09	0.7 ± 0.2	1.1 ± 0.3
CMP-SC	0.41 ± 0.13	0.97 ± 0.15	0.8 ± 0.2	1.3 ± 0.3
CMP-DC	2.7 ± 0.8	0.24 ± 0.1	1.3 ± 0.6	1.2 ± 0.5
S-S	2.6 ± 0.3	0.044 ± 0.005	0.7 ± 0.3	1.1 ± 0.3

## Data Availability

Data are contained within the article and Supplementary Materials.

## Supplementary Materials

The following supporting information can be downloaded at https://www.mdpi.com/article/10.3390/ma17030592/s1, Figure S1: Structure of the two-branch, star-like copolymer m-PEG-P(MMA-ran-DMAEMA)_2_; Figure S2: Viscosity *η_0_* of the A(BC)_2_ solution vs. the mass fraction *x* of A(BC)_2_ dissolved in the solution.; Figure S3: (a) Topographic AFM image of the interfacial region between the CMP substrate (dark region) and the ultrathin polymeric film (brighter region), (b) Exemplificative cross-sectional profile.; Figure S4: Intersections of adjacent peaks and valleys (purple horizontal lines) for a typical MP topographic profile. References [24,26,95,96] are cited in the Supplementary Materials.
