# Supplementary material for: Morphology and Mechanics of Star Copolymer Ultrathin Films Probed by Atomic Force Microscopy in the Air and in Liquid
Source: Materials (Basel). 2024 Jan 25;17(3):592. doi: 10.3390/ma17030592 (PMC10856403; doi:10.3390/ma17030592)
Supplement: Supplementary file 1 [file materials-17-00592-s001.zip › materials-2807155-supplementary.pdf]

Supplementary Information

1. Copolymer mass fraction

Mass fraction of a species, wt%, is a common way to refer to the fraction of a mass of the species divided by the total mass of all species times 100. In our case, 1 mg of $A(BC)_2$ (see Figure S1) is dissolved in chloroform ($CHCl_3$) obtaining solution with concentration c of 1 and 4 mg·ml^{-1}. The chloroform density ρ_{CHCl3} is 1.49 g·ml^{-1}, so the total mass of a ml of solution with c = 1 mg·ml^{-1} is m_{sol} = 1.49 + 0.001 = 1.491 g. The mass fraction of $A(BC)_2$ dissolved in the solution is therefore wt% = $[m_{A(BC)2}\cdot(m_{sol})^{-1}]\cdot 100 = [0.001\cdot(1.491)^{-1}]\cdot 100 = 0.067$ and, accordingly, it will be ≈ 0.268 for c = 4 mg·ml^{-1}.

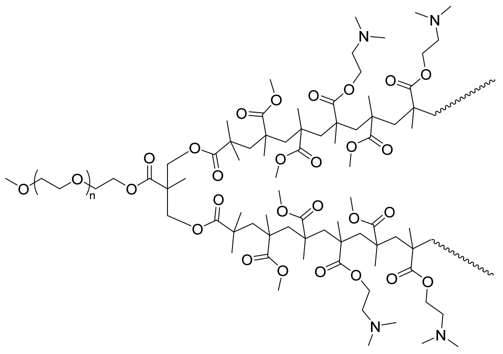

Figure S1. Structure of the two-branches, star-like copolymer m-PEG-P(MMA-ran-DMAEMA)$_2$

2. Density and viscosity of the $A(BC)_2$ solution

The density ρ and the viscosity η_0 of the $A(BC)_2$ solution are unknown, so they are assumed comparable to the ones obtained for MEH-PV [1], a polymer with similar mass M_n, 86 kDa, and polydispersity index PDI, 1.52. The plot η_0 (in cP) vs. wt% is obtained on the basis of data reported in Ref. [1] (see Figure S2).

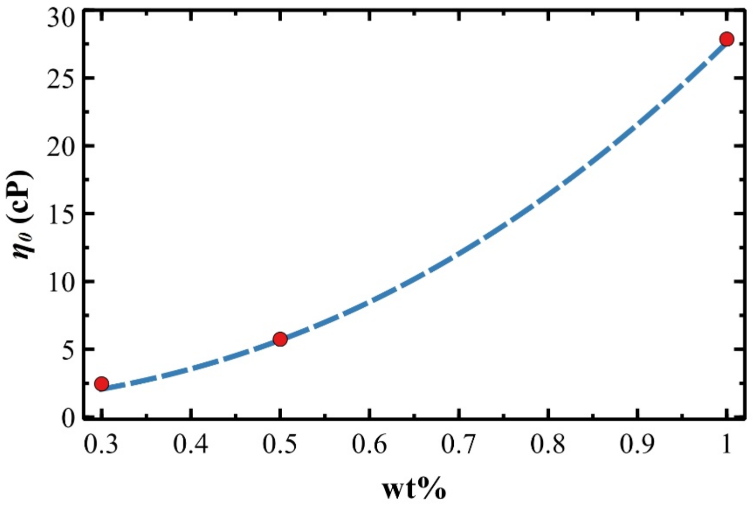

Figure S2. Viscosity η_0 of the $A(BC)_2$ solution vs. the mass fraction x of $A(BC)_2$ dissolved in the solution. The fitting curves following the equation reported in the text (dashed line).

The plot η_0 vs. wt% follows the power law $\eta_0 = A + B \cdot (wt\%)^C$ reported in the literature [1] where the exponent C is ≈ 2.4, a value around 2.5 that is typical for photoresist solutions [2], A is the solvent viscosity fixed to 0.57 cP (i.e. pure $CHCl_3$ at 298 K and wt% = 0) and B ≈ 27 is a proportional constant. The power law well represents experimental data (fitting dashed curve in Figure S2) and η_0 of the $A(BC)_2$ solution is ≈ 0.61 cP for wt% = 0.067, reasonable close to η_0 of pure $CHCl_3$.

3. Thickness of drop-casted ultrathin films

The thickness of drop-casted films obtained by depositing 1 ml of solution on a TEM grid (mesh 300) placed on CMP substrates is measured across the interfacial region film – substrate (see Figure S3).

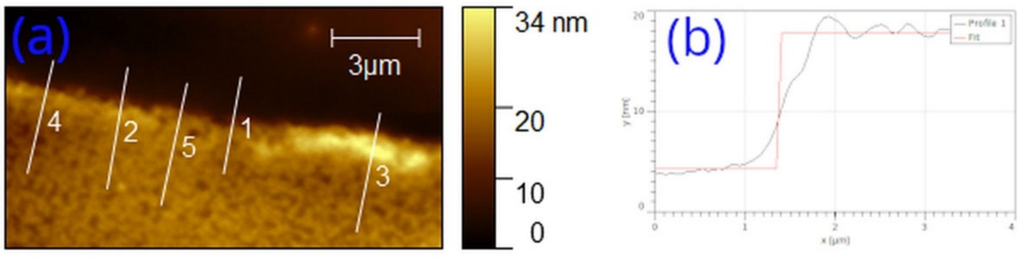

Figure S3. (a) Topographic AFM image of the interfacial region between the CMP substrate (dark region) and the ultrathin polymeric film (brighter region). Five cross-section profiles marked by using the software Gwyddion are reported in the figure (white numbered lines). (b) Exemplificative cross-sectional profile (black line, line 5 in the topographic image) with a step-edge fit to measure film thickness (red line).

Line profiles in Figure S3a, line 5 plotted as exemplificative graph in Figure S3b, shows a single step. The average height of such measured steps (5 in total) is $h_f = (16 \pm 3)$ nm with the absolute error measured by means of the discrepancy between 5 steps, i.e. $(h_{f\text{-}max} - h_{f\text{-}min})/2$.

4. Waviness and roughness of MP and CMP topographic profiles

One dimensional roughness parameters are obtained by splitting a topographic profile into waviness (the low-frequency components) and roughness (the high-frequency components). This procedure depends critically on the cut-off C that is determined by [3]:

$$C = \frac{2\Delta}{\lambda_c} \quad \text{(Eq. S1)}$$

where Δ is the lateral distance between neighbor points in the topographic profile, i.e. the pixel size, and λ_c is the sampling length. There are a number of guidelines available for evaluating λ_c however, as a rule of thumb, it is commonly sets five times the (average) spacing between adjacent peaks and valleys produced by the machining process [4]. AFM images are 10 μm wide with 512 pixels, so Δ = 19.5 nm.

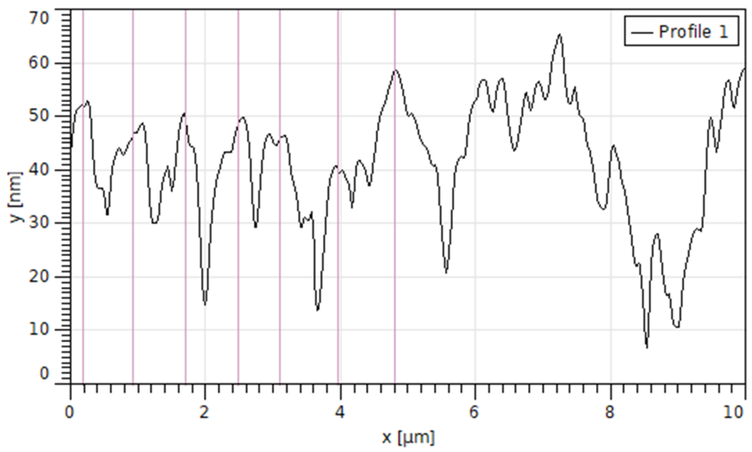

Figure S4. Intersections of adjacent peaks and valleys (purple horizontal lines) for a typical MP topographic profile. To be representative of the entire AFM image, the topographic profile is obtained by averaging 90 adjacent profiles.

As shown in Figure S4, peaks and valleys are present in the topographic profile of the MP surface although it is not periodically spaced. The average distance between two adjacent peaks, also defined as "sample length" [4], is (800 ± 100) nm (see profile intersections in Figure S4) for the MP surface. By following the rule of thumb described above, λ_c is 4000 and C calculated as 0.0098 by using Equation E1.

References

1. Chang, C.-C.; Pai, C.-L.; Chen, W.-C.; Jenekhe, S.A. Spin Coating of Conjugated Polymers for Electronic and Optoelectronic Applications. *Thin Solid Films* **2005**, *479*, 254–260, doi:https://doi.org/10.1016/j.tsf.2004.12.013.

2. Meyerhofer, D. Characteristics of Resist Films Produced by Spinning. *J. Appl. Phys.* **1978**, *49*, 3993–3997, doi:10.1063/1.325357.

3. Palma, D. Re: [Gwyddion-Users] Roughness Parameters Available online: https://sourceforge.net/p/gwyddion/mailman/message/29363255/.

4. Metrology, S. An Introduction to Surface Roughness Measurement Available online: https://www.spectrum-metrology.co.uk/surface-roughness/theory.php.
